# Toxemia of Pregnancy1Read before the section on obstetrics, Buffalo Academy of Medicine, February 26, 1901.

**Published:** 1901-05

**Authors:** Montgomery A. Crockett

**Affiliations:** Buffalo, N. Y.; Gynecologist to the Buffalo General and Erie County Hospitals; adjunct professor of obstetrics and clinical gynecology, University of Buffalo


### TOXEMIA OF PREGNANCY.[1]

By MONTGOMERY A. CROCKETT, M. D., Buffalo, N. Y.,

Gynecologist to the Buffalo General and Erie County Hospitals; adjunct professor of obstetrics and clinical gynecology, University of Buffalo.

THE toxemia of pregnancy has its origin, not in disease, but in the conditions of the gravid state itself; it is an autointoxication resulting from the insufficient elimination or modification of the products of tissue metabolism. This theory of autointoxication was advanced by Bouchard in 1887, and since his time there have been very many investigations to discover the exact nature of the poisons responsible for the affection. Yet to-day, after fourteen years, one rises from the study of the literature of the subject with a feeling of dissatisfaction which is not allayed until one appreciates the limitations of physiologic knowledge.

We know that the body is a vast laboratory and that the metabolic processes continually are producing a quantity and variety of chemical compounds which, after passing through various metamorphoses, are eliminated through the intestines, skin, kidneys, lungs and liver. Like those whose business is the manufacture of high explosives, we become accustomed to the constant presence of the dangerous compounds until, now and then, an accident happens which awakens us to the dangers of the situation. How much of the chemistry of digestion and nutrition still remains unknown to us!

In a recent article (McClure's Magazine, February, 1901,) Professor Remsen of Johns Hopkins University refers to the "awesome

---

1. Read before the section on obstetrics, Buffalo Academy of Medicine, February 26, 1901.

proteids'' and expresses the opinion that they will remain unsolved problems for generations to come; he also says, ''yet everyone who knows about chemistry and physiology knows that these proteids must be understood before we can hope to have a clear conception of the chemical processes of the body.'' In the American Text-book of Physiology, Professor Lusk mentions over twenty principal products of the digestion and putrefaction of proteids and says that their consideration from a chemical standpoint is impossible, for their composition is not known. Have we the right, then, to expect definite statements about autointoxication when one of the chief constituents of the body still remains a mystery?

Then there are the ductless glands about whose internal secretion so little is known beyond the fact that in some way they have an important rôle in the functions of metabolism.

In the intestines a certain amount of bacterial decomposition, especially of carbohydrates, is normal. Where is the boundary line between normal and pathologic bacterial action? Diminished action of the digestive enzymes, deficient absorption from the bowel and a large quantity of proteid material will permit the bacterial decomposition to become so great that products arise which, in some unknown way, disturb even the most distant organs.

The vital processes in the cells themselves are entirely beyond our ken. We note the most wonderful transformations, such as the changing to serum albumin of the peptones and proteoses, substances poisonous to the general economy if directly injected into the circulation. How the cells perform the metamorphoses, what conditions alter their activities and what may be the products of their disordered functions are subjects about which little is known.

Finally, back of all the metabolic processes stands an intricate nervous system, a governing power whose constitution and by-laws not yet have been wholly deciphered. With these chasms in our physiologic knowledge no wonder a discussion of autointoxication brings to light views both indefinite and conflicting. If the mechanism and capability of the human laboratory are so little understood naturally the nature of the output is difficult to determine and, for the present, we must content ourselves with general statements. At all events we know, from observations upon ourselves and animals, that the products of tissue waste, when accumulated within the organism, may produce grave disturbances.

Even a superficial study of the physiology of elimination suggests two ways by which autointoxication may be brought about. First, there may be some impairment in the transforming functions, so that the waste matter is not converted into a chemical composition appropriate to the structure of the eliminative organs; thus intermediate, but normal, products accumulate, and we have toxic symptoms caused by substances which, strictly speaking, are not poisons. Second, through functional insufficiency the exit by way of the excretory organs may be closed and again intoxication from normal products is the result. It must, however, be borne in mind that a disarrangment of metabolism by either of these two ways soon produces alterations of organic function, so that distinctly toxic compounds may appear. Viewing the subject of the toxemia of pregnancy in the light of the above statements we must pass to the consideration of the kidneys, liver and bowels; we omit the skin and lungs because these organs seldom play a primary part in the production of the autointoxication of gravid women.

*The kidneys.*—Eclampsia undoubtedly is an affection of toxic origin and the earlier writers looked to the kidneys as the chief seat of the difficulty. It is well established that in pregnancy the toxicity of the urine is much increased over that passed by the non-gravid woman, and the presence of toxic symptoms is associated with a diminution in the amount of material eliminated by the renal functions. Every physician is familiar with instances of autointoxication caused by renal insufficiency, although the underlying conditions leading to the lessened excretion may have been obscure. Normal urine is a very complex product and our routine clinical examinations are apt to make us forget that it contains many substances for which we seldom or never test. The kidneys eliminate not only the normal end-products of metabolism but also substances occurring in food, but which are not classed as food products. (American Text-Book of Physiology, p. 274, first ed.) The important points to remember are that the symptoms associated with deficient renal action are due to the retention of not one, but many compounds, and that the fault of their non-elimination may not lie with the kidneys.

*Liver.*—This is one of the great transforming organs; it stands between the portal and systemic circulations, protecting the organism from a dangerous accumulation of ammonia compounds by converting them into urea, the bile carries off certain waste substances and in the liver the dextrose is side-tracked as glycogen. Evidently a marked interference with hepatic functions may overwhelm the economy with the precursors of urea. Pinard looks upon the autointoxication of pregnancy as a hepato-toxemia and Bouffe de Sanite

Blaise (Med. Review of Reviews, June, 1899,) mentions the following diagnostic features as indicating deficient hapatic action; progressive decrease in the quantity of urea with increase in the proportion of uric acid and such products of retrograde metamorphosis as leucin and tyrosin; alimentary glycosuria, *i. e.*, if the woman ingests a fixed amount of glucose a portion of it will appear in the urine owing to the impairment of the glycogenic function; there is urobilinuria and the toxicity of the blood-serum is increased.

*Bowels.*—We all are so familiar with instances of toxemia arising from absorption of the products of intestinal dyspepsia and putrefaction, that we need say but little on this part of the subject, except to state that autointoxication from the bowels is one of the great dangers of pregnancy.

Although it is an easy matter thus to divide the subject of toxemia, we soon find that a complication is introduced by the fact that the poisonous products coming from one organ derange another, so that the intoxication becomes cumulative. Such intermediate compounds as uric acid may produce insufficiency of renal action; the intestinal toxins may embarrass the liver and irritate the kidneys. In many cases of toxemia the processes become so involved that the sequence of events is obscured and we must content ourselves with Bouchard's indefinite summing up, that autointoxication is a complex intoxication, resulting from deranged metabolism. On the whole, there is no good reason for assuming that the autointoxication of pregnancy differs in its chemistry and mechanics from the toxemia met with under many other circumstances. We have next to consider the conditions of pregnancy predisposing towards autointoxication.

The demands for the protection and nourishment of the fetus stimulate the anabolic processes of the mother; more food is ingested and the products of digestion and nutrition are increased. During its growth, the fetus adds a larger and larger volume of its own waste to that already present in the maternal circulation. Ordinarily, the burden laid upon the transforming and eliminative organs brings about a physiologic hypertrophy and the woman is protected against harm, but just as there are some hearts which only functionate well as long as they are not exposed to unusual strain, so there are some excretory organs which fail to meet the demands of the hour and such is particularly the case with those women whose elimination is habitually deficient. The cessation of menstration undoubtedly deprives the patient of an important eliminative route. At the very time when excretion becomes so indispensable for the woman's welfare the growing uterine tumor increases intraäbdominal pressure and interferes with the functions of liver, kidneys and bowels. One reason why pregnancy may be regarded as a condition mid-way between health and disease is that it calls for greater elimination and at the same time hinders the process. Many experiments prove the increased toxicity of the blood of pregnancy and the toxicity is greater towards the latter months, both because the fetal excretion is larger and the intraäbdominal pressure greater. So well recognised is this tendency of pregnancy to develop autointoxication that the opinion is growing that almost all the so-called diseases of pregnancy should be regarded as symptoms of toxemia.

*Toxemia and the Hyperemesis of Pregnancy.*—A certain amount of nausea and vomiting is considered to be a part of the physiologic processes of pregnancy, but whenever the gastric disturbances are so severe as to interfere with the welfare of mother or child they can be classed as pathologic. There are no hard and fast lines between simple and pernicious vomiting, the one fades into the other and many of the modern authors are reaching the conclusion that both forms are abnormal phenomena, depending upon the same general causes. All cases of digestive disturbance in pregnancy demand the attention of the physician as he cannot know but that the mildest symptoms may be the precursors of hyperemesis. There are two important elements in the vomiting of pregnancy, one the reflex stimulus and the other the over-susceptible nervous system. Simple vomiting may be but the result of the action of a pregnant uterus upon a nervous system, which has not yet adjusted itself to the gravid state, but irritability of the nerve centers is one of the prominent signs of toxemia and, at least, in severe forms of vomiting, autointoxication is rarely absent. In fact, morning sickness may be one of the factors producing the toxemia. If there be abnormal pelvic conditions, as displacement or inflammation, the reflex stimulation will be all the greater. Intelligent treatment should direct itself both to the general and local conditions. From my own limited experience, I am satisfied that the gastric disturbances of pregnancy are neither frequent nor severe when the patient's elimination is kept up to the standard. It must also be remembered that the toxic elements themselves are sources of irritation and that the vomiting and diarrhea may be efforts at excretion. The long list of therapeutic failures contained in the older text books on obstetrics bears witness to non-appreciation of the relation between toxemia and pernicious vomiting.

*Toxemia and Eclampsia.*—The weight of evidence today is in favor of the toxic etiology for eclampsia. There have been attempts to fasten a microbe upon this affection, but without much success. When we recollect that toxemia is a favorable condition for infection the suspicion arises that the presence of bacteria is a result and not a cause. The symptoms of headache, mental irritability, tinnitus, etc., which constitute what is known as the pre-eclamptic stage, are the typical symptoms of toxemia and it is a striking fact that eclampsia is rare among those patients whose excretion is sufficient.

Neuralgia, ptyalism, insomnia, herpes zoster, sweating, pruritus and melancholia are other disorders of pregnancy which may be caused by toxemia and which I will dismiss without comment.

If these views regarding the relation between autointoxication and gestation be but partially true, the importance of urinalysis during pregnancy cannot be overestimated. A sample from the twenty-four hour urine should be examined every two weeks; oftener when there are least indications of disturbance. The patient herself should be directed to note striking variations in the amount of urine passed. Many patients do not appreciate the importance of using a strictly clean bottle; a few days ago I received urine showing a specific gravity of 1040 and the presence of sugar. The glycosuria was cured by prescribing a clean bottle in place of the one formerly containing Mellin's food. A great many pregnant women are troubled with leucorrhea, enough to contaminate the urine and give a test for albumin; therefore it is well to direct the patient to take a vaginal douche and introduce a tampon of absorbent cotton just before urinating; a string tied to the tampon makes removal easy. The estimation of the percentage of urea is the most important part of the urinalysis, not that the urea is a serious poison, but the degree of its elimination is a valuable index of renal excretion. In the urine of pregnancy the urea varies from 1.4 to 2 per cent. (E. P. Davis); less than 1 per cent. indicates danger.

Albumin is present in 5 per cent. of all cases of pregnancy, and in a large proportion of the patients does not appear until the latter months of gestation. Allbutt does not believe in pressure as a cause of albuminuria on the ground that the renal veins are not easily pressed upon and that albuminuria often is absent when the pressure is greatest; he thinks that the poisons absorbed from the intestinal tract are responsible for the kidney disturbance. Under all circumstances albuminuria calls for treatment as there has been established a definite relation between this condition and lessened development of the fetus; albuminuria frequently is associated with hemorrhages into the placenta, which may be extensive enough to destroy the fetus. It is likely that the toxins are the underlying causes of both the renal disturbance and the injury to the child. Jewett (*Brooklyn Medical Journal*, August, 1899,) says that albumin is present in between 80 and 90 per cent. of eclamptic patients previous to the first convulsion and that eclampsia is rare when the daily amount of urine is three pints. He also states that a large amount of urine, say 70 ounces in twenty-four hours, will eliminate the poisons even when the tests show albuminuria and diminished urea. The bearing of these facts upon the importance of measuring and fully examining the urine of pregnant women is obvious. It is unfortunate that we have no clinical test by which we can estimate the toxicity of the urine, for a marked diminution in toxicity would be a valuable warning of approaching danger.

The treatment of toxemia is based upon ordinary common sense. The manufacture of the poisons is lessened by restricting the diet; in bad cases limiting it to milk; in some instances we must strike at the source of all the evil and empty the uterus. Elimination is promoted by stimulating the excretory powers of the bowels, skin and kidneys. The great value of normal salt solution in all cases of toxemia should not be passed over without mention; in mild forms of autointoxication the solution may be introduced per rectum; in severe, hypodermoclysis should be employed Salt solution has given very favorable results in eclampsia, in which affection it is most effective when used intravenously, preceded by bleeding in appropriate cases. The saline infusion not only dilutes the poisons but promotes diuresis; even a few ounces of salt solution may cause a copious flow of urine.

In conclusion, we wish to state that the theory of autointoxication as applied to pregnancy has led to a much more intelligent understanding of the disorders of pregnancy, and that the physician who acts on this theory will not only make his patient more comfortable, but protect her from serious danger. If the pregnant woman would place herself under the care of an able physician from the very beginning of gestation, and if the medical attendant performed his whole duty, the socalled diseases of pregnancy would show a marked diminution in frequency. It is true, we know little about the toxic products themselves, but we understand enough of the general processes of metabolism to appreciate how the poisons may arise, to recognize the symptoms which they produce and to work in harmony with nature for their removal.

